# Improved Surface-Based Registration of CT and Intraoperative 3D Ultrasound of Bones

**DOI:** 10.1155/2018/2365178

**Published:** 2018-06-03

**Authors:** Zian Fanti, Fabian Torres, Eric Hazan-Lasri, Alfonso Gastelum-Strozzi, Leopoldo Ruiz-Huerta, Alberto Caballero-Ruiz, F. Arámbula Cosío

**Affiliations:** ^1^Instituto de Investigaciones en Matemáticas Aplicadas y en Sistemas, Universidad Nacional Autónoma de México, Ciudad Universitaria, Circuito Escolar S/N, 04510 CDMX, Mexico; ^2^Instituto de Ciencias Aplicadas y Tecnología, Universidad Nacional Autónoma de México, Ciudad Universitaria, Circuito Exterior S/N, 04510 CDMX, Mexico; ^3^Instituto Nacional de Rehabilitación, Calzada México Xochimilco No. 289, Colonia Arenal de Guadalupe, 14389 CDMX, Mexico; ^4^National Laboratory for Additive and Digital Manufacturing, Mexico

## Abstract

The intraoperative registration of preoperative CT volumes is a key process of most computer-assisted orthopedic surgery (CAOS) systems. In this work, is reported a new method for automatic registration of long bones, based on the segmentation of the bone cortical in intraoperative 3D ultrasound images. A bone classifier was developed based on features, obtained from the principal component analysis of the Hessian matrix, of every voxel in an intraoperative ultrasound volume. 3D freehand ultrasound was used for the acquisition of the intraoperative ultrasound volumes. Corresponding bone surface segmentations in ultrasound and preoperative CT imaging were used for the intraoperative registration. Validation on a phantom of the tibia produced encouraging results, with a maximum mean segmentation error of 0.34⁡mm (SD=0.26⁡mm) and a registration accuracy error of 0.64⁡mm (SD=0.49⁡mm).

## 1. Introduction

The use of image-guided surgery (IGS) systems has expanded significantly for more than 20 years, in the number of procedures performed and the variety of clinical applications, in part due to the continuous advancement of computer power and medical imaging systems (as well as tracking systems, registration methods, software development frameworks, and visualization techniques). The main clinical applications of IGS systems in orthopedics are pedicle screw insertion, hip replacement, knee replacement, and fracture alignment [1].

Scheufler et al. [2] reported the results of image-guided instrumentation of the cervical, upper, and midthoracic pedicles. The study considers the insertion of 248 pedicle screws and reports safe and highly accurate results. Jeswani et al. [3] reported the evaluation of CT image-guided navigation of pedicle screws in small thoracic pedicles. The results showed that image-guided navigation allows for safe, effective, and accurate instrumentation of small (≤3⁡mm) to very small (≤2⁡mm) thoracic pedicles. Deep et al. [4] reviewed the literature on computer-assisted orthopedic surgery (CAOS) for knee and hip arthroplasty; the authors conclude that CAOS systems have gained a pivotal role in lower limb arthroplasty. The use of image-guided surgery (IGS) systems in trauma has been explored for the last eight years approximately, and several methods and systems have been developed with various levels of success [5].

The recent development of minimally invasive arthroscopic procedures for fracture fixation in long bones has substantially improved the accuracy of the procedures with shorter patient recovery times. The success of the surgery depends heavily on the skill and experience of the surgeon [6]; however, the use of CAOS systems in fracture fixation performed with arthroscopic procedures offers an improved visualization of the surgical site with position feedback [7]. It has also been reported that the use of CAOS, in arthroscopic fracture reduction procedures, improves the accuracy of the surgery and achieves a shorter and more effective patient recovery [8]. Buschbaum et al. [9] reported the development of a virtual environment which helps the surgeon with planning optimal reduction paths for femoral fractures. Weil et al. [10] reviewed the literature on the evolution of image-guided iliosacral screw placement, for the reduction of pelvic ring fractures. 3D image-guided surgery is faster and more accurate and uses less X-rays than conventional fluoroscopy-guided surgery. The authors stress the challenges of platform interoperability, learning times, and cost reductions.

The main stages of most CAOS systems are preoperative image acquisition (usually computed tomography) of the anatomic region of interest, computer-assisted modeling and surgery planning, intraoperative image registration, and finally surgery execution with some computer assistance based on the surgical plan. Intraoperative registration is a critical process of most CAOS systems, given that registration accuracy determines the precision of the surgical visualization, planning, and navigation with respect to the patient on the operating table [11].

Several methods have been developed for intraoperative registration of preoperative CT volumes for CAOS, with fiducial-based registration as the most widely used. Preoperatively, fiducial points are annotated in the medical images or graphic models of the bones, and intraoperatively, the same points are located in the bones of the patient using a navigated tool [12]. Usually only a few anatomical landmarks can be reliably selected; therefore, artificial fiducial markers have been used. The marker is surgically attached directly to the bone of the patient before the preoperative study is acquired. This is an accurate registration approach; however, it increases the complexity of the surgical procedure and the risk of complications due to the invasiveness required to reach each fiducial point.

In order to minimize the invasiveness of the registration process while maintaining high accuracy, image-based methods have been developed. Image-based registration has clear advantages: no need for artificial fiducials, therefore, limiting bone exposure; high accuracy can be obtained through registration of large surface areas. Fluoroscopy-based registration has been extensively used [7, 13, 14]. However, fluoroscopes are large and expensive and expose the patient and the surgical staff to ionizing radiation. On the other hand, ultrasound is a convenient and safe intraoperative imaging modality; it is portable, low cost, and real time. Its main limitations are low signal to noise ratio due to speckle, user-dependent acquisition and interpretation, and inability to penetrate bones.

Previous studies have shown that the use of intraoperative ultrasound is feasible to achieve errors that satisfy the accuracy requirements of surgery. Herring et al. [15] reported the use of spine phantoms submerged in water to locate fiducial points in ultrasound images. Submillimetric accuracy was reported for the registration of the fiducial points with the spine phantom. Ionescu et al. [16] reported one of the first registration methods based on the segmentation of the bone cortical in ultrasound images. Yan et al. [17] achieved registration errors of less than 2.0⁡mm in transpedicular screw insertion in porcine cadaver experiments and also less than 1.0⁡mm of error in phantom experiments.

Most methods for image-based registration of CT and ultrasound can be classified as intensity-based or surface-based methods. Surface-based registration requires a process of feature extraction in both modalities, and registration accuracy is then affected by feature extraction. Meanwhile, intensity-based registration methods optimize a similarity (objective) function. The following groups, Penney et al. [18], Winter et al. [19], and Gill et al. [20], developed different intensity-based registration methods and reported registration errors under 2.0⁡mm. The most important difference between both registration approaches (intensity or surface-based) is its suitability for a specific surgical procedure.

In surface-based registration methods, the manual, or automatic, segmentation of the bone surface in the intraoperative ultrasound images is necessary. Kowal et al. [21] developed an early automatic segmentation method for 2D ultrasound images of bones, based on pixel intensity and position, since bones usually appear bright and are located at the top of the ultrasound images. The method is sensitive to image contrast, having an average segmentation error of 0.42⁡mm (SD=0.19⁡mm).

Beek et al. [22] reported a method for surface-based registration during scaphoid fracture reduction surgery, 3D ultrasound images are segmented semiautomatically with the annotation of 10 seed points in the cortical of the bones, registration is performed using iterative closest points, and a segmentation error of 0.56⁡mm (SD=0.08⁡mm) was reported. Hacihaliloglu et al. [23] developed a bone segmentation method for 3D ultrasound images, based on the Log-Gabor filters and phase coherence, and a segmentation error of 0.28⁡mm (SD=0.24⁡mm) was reported for validation in bone phantoms.

In this work, is reported a new method for the automatic segmentation of the bone cortical in 3D ultrasound imaging. The method is based on a voxel classifier, trained with features obtained from the eigenanalysis of the Hessian matrix corresponding to every voxel in the ultrasound volume. The segmentation of the bone surface in an ultrasound volume is subsequently used for intraoperative registration of the corresponding surface segmented in a CT volume. In the following sections, are reported the acquisition of 3D freehand ultrasound volumes of long bones, the segmentation of the bone surface (cortical), and the registration of a preoperative CT volume. Experimental validation was performed on a phantom of the tibia.

## 2. Bone Segmentation and Registration Methods

### 2.1. Freehand Acquisition and Reconstruction of 3D Ultrasound Volumes

3D freehand ultrasound images were acquired with a conventional 2D scanner (Aloka 1000, Hitachi Aloka Medical America, Inc.) using a 7.5 MHz probe. An optical tracker (Polaris Spectra, Northern Digital, Inc.) was used to navigate the ultrasound probe. Ultrasound B-mode images were continuously acquired using a frame grabber (Epiphan Systems Inc.). For the reconstruction of the 3D ultrasound volumes, the probe was calibrated using the cross-wire methodology [24], and 3D volumes were reconstructed using the pixel-based method reported in [25]. Full details of the acquisition and reconstruction of 3D freehand ultrasound images have been published elsewhere [26, 27].

### 2.2. Segmentation of the Bone Surface in 3D Ultrasound

The segmentation method has two main stages: bone surface enhancement, using feature extraction based on differential geometry, and surface segmentation using a Bayes classifier and a region growing algorithm. Bone surfaces in an ultrasound image are identified as bright regions above dark regions caused by the acoustic shadows produced by the bone. High-intensity echoes produced by the bone surface can be observed in ultrasound images as bright lines with a width of 2–4 mm. Jain and Taylor [28] have shown that a good estimate of the bone cortical in an ultrasound image lies at the center line of the bright echo line. The center line of the ultrasound echoes corresponds to the maximum principal curvature in the, orthogonal, gradient direction. This allows for the detection of the bone cortical using a method based on ridge detection as described below.

#### 2.2.1. Bone Surface Enhancement in 3D Ultrasound

The second derivatives, in each direction around a voxel in an ultrasound volume, provide information about the type of geometric shape to which the voxel belongs. The geometric shapes could be blobs, tubes, or surfaces [29]. Shape information was obtained from the principal component analysis of the Hessian matrix calculated on each voxel *I*(*x*, *y*, *z*) in the volume. The Hessian matrix (2) was constructed with the second partial directional derivatives, which are approximated with the convolution of every voxel in the volume *I*(*x*, *y*, *z*) with a Gaussian kernel (1) in each direction,

(1) $$\begin{array}{c} G\left(x,\;\sigma \right)=\frac{1}{{\left(\sqrt{2\pi }\sigma \right)}^{n}}\exp-\frac{{\left\|x\right\|}^{2}}{2\sigma^{2}}, \end{array}$$

where *σ* is the scale factor of the kernel. The size of the kernel is determined as three times *σ*. The Hessian matrix of a voxel is a symmetric matrix that contains the partial derivatives in all the possible directions as shown in the following equation:

(2) $$\begin{array}{c} H=\left[\begin{array}{ccc} \partial_{xx}I & \partial_{xy}I & \partial_{xz}I\\ \partial_{yx}I & \partial_{yy}I & \partial_{yz}I\\ \partial_{zx}I & \partial_{zy}I & \partial_{zz}I \end{array}\right], \end{array}$$

where each ∂_*ij*_ *I* represents the second order partial derivative of the voxel in the directions *i* and *j*. The principal component analysis of the Hessian matrix results in three eigenvalues *λ*_1_, *λ*_2_, and *λ*_3_ and the corresponding eigenvectors *v*_1_, *v*_2_, and *v*_3_, for each voxel in the ultrasound volume. If *λ*_1_ ≥ *λ*_2_ ≥ *λ*_3_, there is a set of conditions that determines the membership of a voxel to a certain type of geometric shape which can be tubular, spherical, or a surface. The conditions are shown in Table 1.

Based on the conditions shown in Table 1, voxels that belong to a surface were chosen and assigned a new value given by (3). This highlighted all the voxels that had a high probability of belonging to a surface [29],

(3) $$\begin{array}{c} S_{\text{sheet}}\left(\lambda_{1},\;\lambda_{2},\;\lambda_{3}\right)=\left\{\begin{array}{ll} \left|\lambda_{3}\right|\cdot \omega \left(\lambda_{2};\;\lambda_{3}\right)\cdot \omega \left(\lambda_{1};\;\lambda_{3}\right), & \lambda_{3}<0\\ 0, & \text{otherwise}, \end{array}\right. \end{array}$$

where

(4) $$\begin{array}{c} \omega \left(\lambda_{i};\;\lambda_{j}\right)=\left\{\begin{array}{ll} {\left(1+\frac{\lambda_{i}}{\left|\lambda_{j}\right|}\right)}^{\gamma }, & \lambda_{j}\leq \lambda_{i}\leq 0\\ {\left(1-\alpha \frac{\lambda_{i}}{\left|\lambda_{j}\right|}\right)}^{\gamma }, & \frac{\left|\lambda_{j}\right|}{\alpha }>\lambda_{i}>0\\ 0, & \text{otherwise}. \end{array}\right. \end{array}$$

Equations (3) and (4) represent the condition that one voxel belongs to a surface. The condition *λ*_3_ ≪ *λ*_2_≃⁡*λ*_1_≃⁡0 was expressed as *λ*_3_ ≪ *λ*_2_≃⁡0 and *λ*_3_ ≪ *λ*_1_≃⁡0; those inequalities are described by (4) where the value of *ω* decreases with the deviation from the condition *λ*_1_≃⁡0. As recommended by Sato et al. [29], we choose the values *α*=0.5 and *γ*=1.

#### 2.2.2. Bone Surface Extraction in 3D Ultrasound

A bone segmentation algorithm based on a Bayes classifier was developed. The classifier was trained with features of the voxel intensity and the first, second, and third moments of the enhanced voxel values previously calculated. Three classes were considered for training: bone, soft tissue, and acoustic shadow produced by the bone. For each voxel, a feature vector was constructed as follows: $\vec{x}\left(x,\;y,\;z\right)=\left[I\left(x,\;y,\;z\right),\;E\left(x,\;y,\;z\right),\;\mu ,\;\sigma^{2},\;\gamma \right]$, where *I*(*x*, *y*, *z*) and *E*(*x*, *y*, *z*) are the original image and the enhanced image values at a specific position; *μ*, *σ* ^2^, and *γ* are the mean, variance, and skewness in a (9 × 9 × 9) window centered in *E*(*x*, *y*, *z*). For each vector $\vec{x}\left(x,\;y,\;z\right)$, the probability of membership to each class, bone, tissue, and shadow, was estimated using the Bayes posterior probability defined in the following equation:

(5) $$\begin{array}{c} P\left(C_{k}\left|\vec{x}\right.\right)=\frac{P\left(\vec{x}\left|C_{k}\right.\right)\cdot P\left(C_{k}\right)}{P\left(\vec{x}\right)}, \end{array}$$

where *P*(*C*_*k*_) is the a priori probability for each class obtained from the training sample proportions and $P\left(\vec{x}\right)$ is the a priori probability of vector $\vec{x}$. $P\left(\vec{x}\left|C_{k}\right.\right)$ is the conditional probability of $\vec{x}$ given *C*_*k*_. To estimate $P\left(\vec{x}\left|C_{k}\right.\right)$, we assumed a normal distribution for each class (bone, tissue, and shadow) as defined in the following equation [30]:

(6) $$\begin{array}{c} G_{N}\left(\vec{x}\right)=\frac{1}{2\pi^{N/2}\sqrt{\left|\Sigma \right|}}\exp\left\{-\frac{1}{2}{\left(\vec{x}-\vec{\mu }\right)}^{T}\Sigma^{-1}\left(\vec{x}-\vec{\mu }\right)\right\}, \end{array}$$

where ∑ is the covariance matrix and $\vec{\mu }$ is the mean of a set of vectors $\vec{x}$ that belong to the class. Substituting (6) in (5) and discarding $P\left(\vec{x}\right)$, we can calculate a discriminant function *Y*_*k*_:

(7) $$\begin{array}{c} Y_{k}\left(\vec{x}\right)=-\frac{1}{2}{\left(\vec{x}-{\vec{\mu }}_{k}\right)}^{T}\Sigma_{k}^{-1}\left(\vec{x}-{\vec{\mu }}_{k}\right)-\frac{1}{2}\ln\left|\Sigma_{k}\right|+\ln\left(P\left(C_{k}\right)\right). \end{array}$$

Equation (7) allowed us to classify each voxel in an ultrasound volume as follows: for each voxel, we calculate the corresponding vector $\vec{x}$ and evaluate (7) for each of the classes: tissue, bone, and shadow. A voxel was labeled as bone (i.e., labeled as “1”) if $Y_{\text{bone}}\left(\vec{x}\right)>Y_{\text{tissue}}\left(\vec{x}\right)$ and $Y_{\text{bone}}\left(\vec{x}\right)>Y_{\text{shadow}}\left(\vec{x}\right)$; otherwise, it was labeled as background (i.e., labeled as “0”).

The mean and covariance of each class (bone, tissue, and shadow) were estimated on a small training volume (150 × 150 × 150) which should include voxels of the three classes. To produce one complete surface with a minimum of holes, a region growing process was also applied [31]. Seed points were selected from the 0.01 percent of the previously classified bone voxels with the highest probability. Starting from these seed points, the bone surface was segmented considering all the neighboring voxels previously classified as bone.

### 2.3. Intraoperative Registration of CT Volumes

The bone surface (cortical) was segmented manually in the preoperative CT volume, and automatically in the intraoperative ultrasound volume, using the method reported in the previous section. From each segmentation, the corresponding mesh was generated using Marching Cubes [32], and each mesh has around 2.5 × 10^5^ vertices. Registration was performed in two stages. First, a few points (three or four) are selected manually with a coarse accuracy, in both meshes, to have an initial rough alignment required by the iterative closest point (ICP) method. Then a subset of 2000 points was randomly acquired from both meshes. Mesh registration was performed using iterative closest points [33]. The software was developed using 3D Slicer [34] with specific modules developed for this research using ITK (http://www.itk.org) and VTK (http://www.vtk.org).

## 3. Results and Discussion

**Figure 1:**
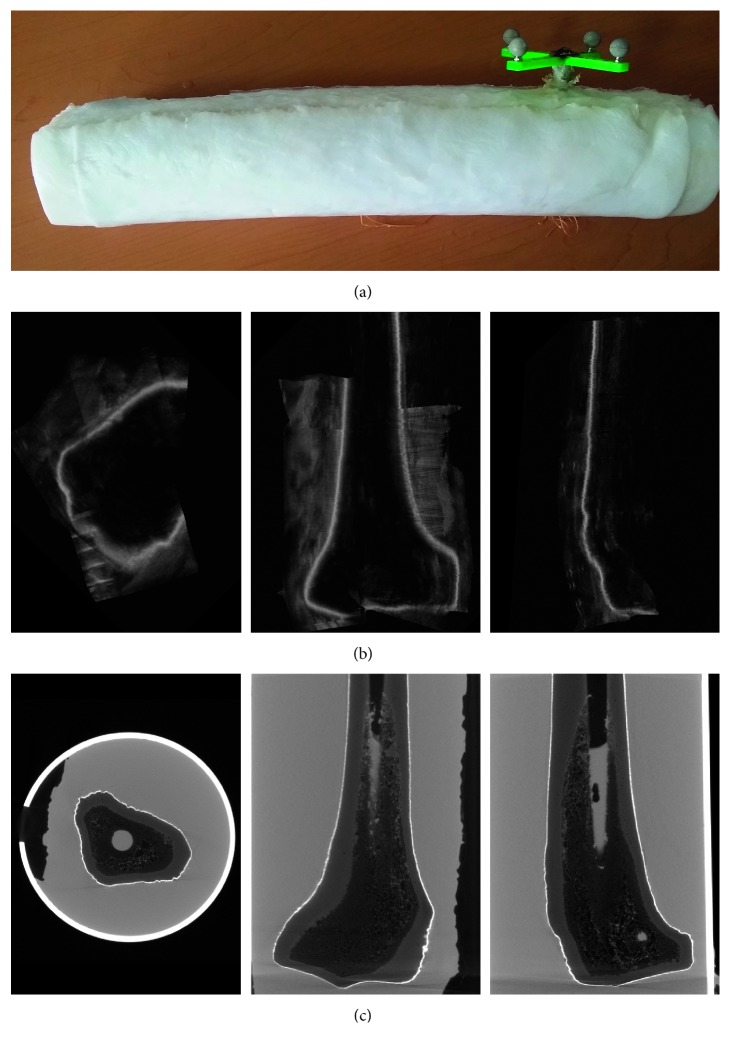
Polyvinyl alcohol (PVA) phantom used in this study. (a) PVA phantom with the tracking tool attached. (b) Three orthogonal views from a 3D ultrasound volume of the phantom acquired, with the 3D freehand ultrasound technique. (c) Three orthogonal views from the CT volume of the phantom.

The accuracy of the bone segmentation and registration was evaluated on a realistic phantom of the tibia, which was constructed with a synthetic tibial bone (ERP #1117-42, Sawbones Inc., Vashon, WA, USA) immersed in a hydrogel made of polyvinyl alcohol (PVA) diluted in 95% of water. It has been shown that PVA resembles the appearance and the mechanical properties of soft tissue on ultrasound images [35]. A passive tracking tool was firmly attached to the distal end of the tibia phantom.  Figure 1(a) shows a photo of the PVA phantom of the tibia. The dimensions and shape of the tibia phantom were accurately measured using microCT.

### 3.1. CT Image Acquisition

MicroCT (Nikon Metrology XTH 225) with a 2048 × 2048 pixel matrix was used to scan the phantom of the tibia including the tracking tool; imaging settings were 220 kV, 61 *µ*A, 3142 projections, and one image per projection, and no physical filter was used. The final volume dimensions were 1042 × 1250 × 3201 voxels in (*x*, *y*, *z*) axes, respectively, with an isometric voxel size of 0.115 mm. The acquired CT volume had a very high resolution that helps improving the accuracy measurements due to a small resolution error (0.057⁡mm) in the CT data.  Figure 1(c) shows three orthogonal views of the acquired CT volume of the phantom.

### 3.2. 3D Freehand Ultrasound Image Acquisition

3D freehand ultrasound images were acquired from the tibia phantom, and the volume was reconstructed as described in Section 2.1. The origin of our coordinate frame was located at the center of one of the reflecting spheres of the tracking tool attached to the tibial bone, shown in Figure 1(a), and the voxel size for the reconstruction of the ultrasound volume was the same of the CT: 0.115 mm. The phantom was scanned with the ultrasound probe using the same water-based gel used for clinical ultrasound scans. The phantom was scanned at different sections always including the tibial plateau. In Figure 1(b), are shown three orthogonal views of the acquired ultrasound volume of the phantom.

### 3.3. 3D Ultrasound Segmentation

A small ultrasound volume (150 × 150 × 150 voxels) was acquired from the test phantom, for training of the segmentation method. Bone, tissue, and shadow regions were manually annotated. The corresponding voxel feature values were stored as the training sample for the Bayes classifier described in Section 2.2. This training set was used for all the experiments reported below, and the training process was performed only once for all experiments.

**Figure 2:**
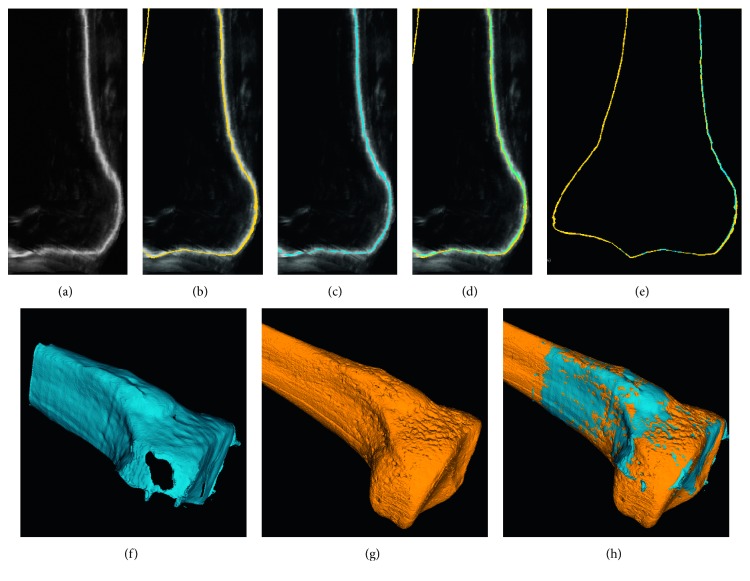
Results of the segmentation process. (a) One slice of the ultrasound volume of the tibia phantom. (b) Same slice in A with the CT segmentation overlapped. (c) Same slice in A with the result of the ultrasound segmentation overlapped. (d) Same slice in A with the result of the ultrasound and CT segmentations overlapped. (e) Ultrasound and CT reference segmentations overlapped. (f) 3D reconstruction of the resulting ultrasound segmentation of one experiment. (g) 3D reconstruction of the CT reference segmentation. (h) 3D reconstructions ultrasound and CT overlapped.

The accuracy of the bone segmentation of the intraoperative ultrasound volumes was measured using as a reference the manual segmentation of the bone in the high-resolution CT data. This segmentation was approved by an expert orthopedic surgeon. As previously mentioned, all the intraoperative ultrasound images were acquired with the origin of the navigation reference frame located at the tracking tool attached to the plastic bone. Since the position of the tracking spheres in the corresponding CT volume was determined, the CT and the ultrasound images were accurately registered. All the remaining errors between the bone surface in CT and ultrasound were mainly due to segmentation errors of the bone cortical in the ultrasound volume (as illustrated in Figure 2).

**Figure 3:**
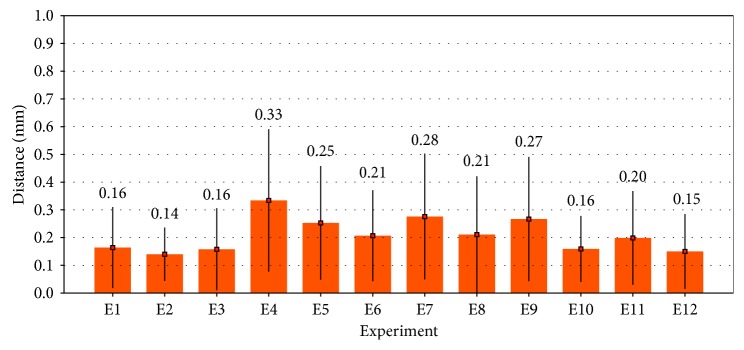
Distribution of the segmentation errors. The height of each bar represents the mean distance error, and the black line represents the standard deviation for each experiment. The mean error values are shown on top of each bar.

Twelve acquisitions of 3D freehand ultrasound volumes were performed at different sections of the phantom of the tibia, which was located each time at different positions and orientations on the experiment table. The bone cortical on each volume was automatically segmented, and the corresponding mesh was constructed.  Table 2 summarizes the mean distances of each node of the ultrasound mesh to the nearest node of the reference CT mesh. As can be observed in Table 2, the maximum mean segmentation error was 0.334 mm (SD=0.257⁡mm) for experiment four, and Figure 3 shows graphically the segmentation errors. The overall mean segmentation error for the twelve experiments performed was 0.21 mm (SD=0.061⁡mm). The average processing time per experiment including volume reconstruction and volume segmentation was about five minutes. Holes in the segmentation, due to shadows in the acquisition of bone ultrasound, were not taken into account.

### 3.4. Intraoperative Registration

Twelve registration experiments were performed; on each one of them, the mesh obtained from the manual segmentation of the bone surface in the CT images of the phantom was registered with the tibia phantom located at different positions on the experiment table; using the method described in Section 2.3, the errors of the registration process were measured as follows.

**Figure 4:**
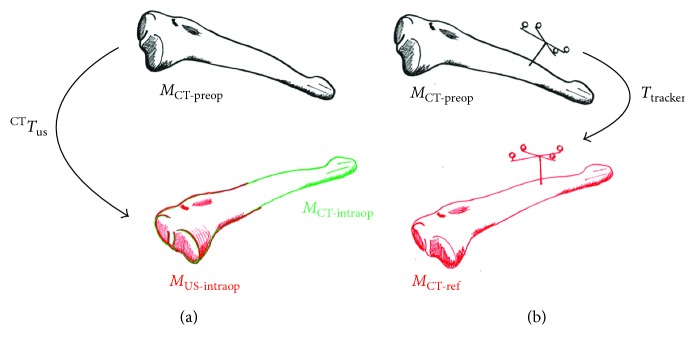
(a) Registration of the preoperative CT mesh (*M*_CT‐preop_) and the intraoperative ultrasound mesh (*M*_US‐intraop_); the registered CT mesh (*M*_CT‐intraop_) is shown in green. (b) Registration of the preoperative CT mesh (*M*_CT‐preop_) using the tracking tool to achieve an accurately registered mesh *M*_CT‐ref_.

For each position of the phantom on the table, a 3D freehand ultrasound volume of a different section of the tibia was acquired. The bone surface in the ultrasound volume was automatically segmented using the method described in Section 2.2.2; from the resulting segmentation, a mesh called *M*_US‐intraop_ was constructed. The mesh constructed from the manual segmentation of the bone surface in the CT images of the phantom, called *M*_CT‐preop_ was registered with *M*_US‐intraop_ using iterative closest points as described in Section 2.3. The resulting registered CT mesh was called *M*_CT‐intraop_ (Figure 4(a)). In order to have a reference to measure the registration error, *M*_CT‐preop_ was also registered with the phantom on each position using the transformation given by the optical tracker, and this accurately registered CT mesh was called *M*_CT‐ref_ (Figure 4(b)). The accuracy of each registration experiment was then measured between the registered CT mesh (*M*_CT‐intraop_) and the reference CT mesh of the tibia phantom (*M*_CT‐ref_), as described below.

**Figure 5:**
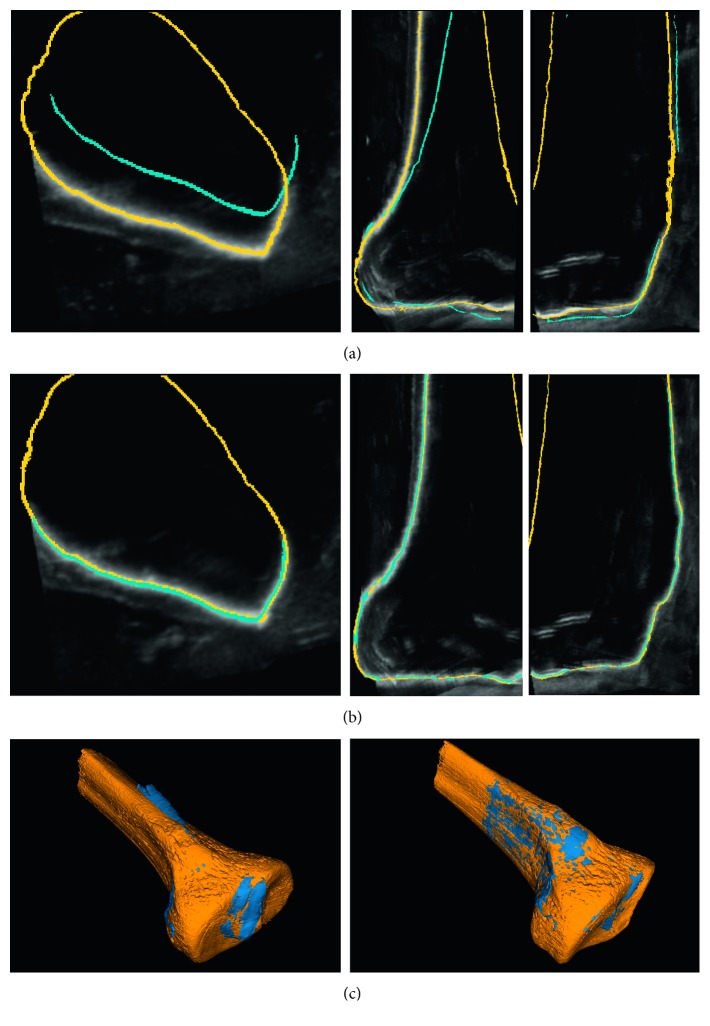
(a) Orthogonal views of the ultrasound volume with the unregistered ultrasound mesh (shown in blue) and registered reference CT mesh (shown in yellow). (b) Orthogonal views of the ultrasound volume with the registered ultrasound and CT meshes overlapped. (c) Ultrasound (blue) and CT (yellow) 3D meshes before (left) and after (right) registration.

**Figure 6:**
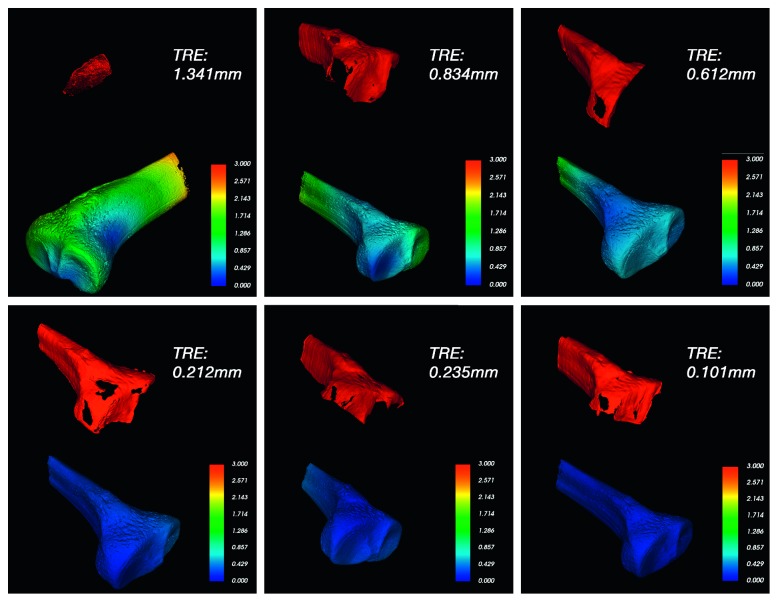
Results of six different experiments. The result of the bone segmentation in the intraoperative ultrasound is shown in red. The registered CT mesh is shown with a false color scale illustrating the target registration error (in mm), for each point on the surface of the CT. The mean target registration error, of each experiment, is shown as TRE on the top-right corner of each subfigure.

**Figure 7:**
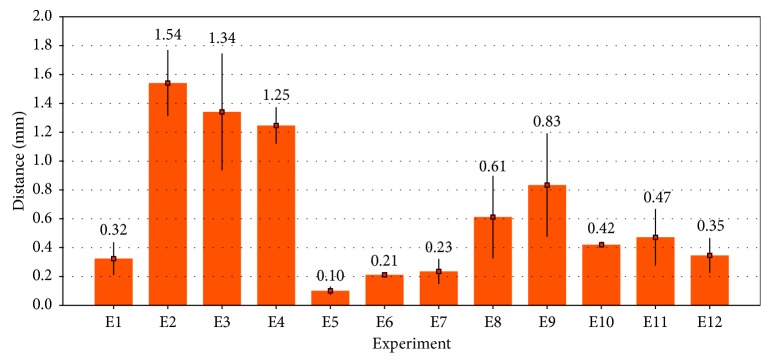
Distribution of the registration errors. The height of each bar represents the mean error, and the black line represents the standard deviation for each experiment. The mean error values are shown on top of each bar.

For each experiment, the same preoperative CT mesh *M*_CT‐preop_ was registered against the tibia phantom by two independent methods: *M*_CT‐intraop_ registered with the method reported in Section 2.3 and *M*_CT-ref_ exactly registered using the tracking tool. Target registration errors (TREs) of each experiment were measured as the mean distance between all corresponding vertices in both registrations.  Figure 5 shows the results of one experiment before (Figure 5(a)) and after (Figure 5(b)) registration, the reference mesh is shown in yellow, the ultrasound generated mesh is shown in blue, and both meshes are shown overlapping on one slice of the ultrasound volume.  Figure 6 shows the errors for six experiments, showing the ultrasound registered mesh in red and the TRE in false color on the reference mesh. The TRE values for all the experiments are reported in Table 3 and are shown graphically in Figure 7. The average time taken by the registration process was approximately two minutes.

### 3.5. Discussion

A fully automatic 3D method was developed for the segmentation of the cortical bone in 3D ultrasound images, acquired with the 3D ultrasound freehand technique, which enables the acquisition of large volumes. The eigenvalues, corresponding to the principal component vectors, of the 3D Hessian matrix of each voxel in an ultrasound volume, were used to enhance the bone surface following the work of Sato et al. [29]. A Bayes classifier was trained with five features: the original and enhanced voxel intensity values, as well as the first, second, and third moments of the enhanced voxel values. Validation of the segmentation method on a realistic phantom of the tibia immersed in PVA, allowed for accurate estimates of the segmentation errors, since PVA has mechanical properties similar to those of tissue in ultrasound imaging. The acquisition of ultrasound images was performed with the same water-based gel used clinically.

Segmentation errors were measured as the distances between all the points of the segmented ultrasound mesh and the nearest points in the reference CT mesh (Section 3.3).  Table 2 summarizes the segmentation errors obtained for twelve different ultrasound volumes of the same phantom (approximate volume size of 850 × 850 × 850 voxels that corresponds to 10 × 10 × 10 cm). A maximum mean error of 0.334⁡mm (SD=0.257⁡mm) for experiment four is shown. Figure 3 shows the distribution of all the mean error values, and the overall mean for the twelve segmentation experiments performed was 0.21⁡mm (SD=0.061⁡mm). These results show improvement over previously reported methods for the segmentation of bones in 3D ultrasound. Kowal et al. [21] reported the automatic segmentation of bone contours in 2D ultrasound images, with a mean segmentation error of the bone contour lines of 0.42⁡mm (SD=0.19⁡mm). Beek et al. [22] reported an IGS system for the fixation of scaphoid fractures based on preoperative CT and intraoperative ultrasound images. A semiautomatic method was developed for the segmentation of bone contours in 2D ultrasound. Validation was performed on plastic phantoms of the scaphoid bone immersed in water. The authors reported a mean segmentation error of 0.56⁡mm (SD=0.08⁡mm). Hacihaliloglu et al. [23] reported an automatic method for bone segmentation based on Log-Gabor filters and phase coherence. A mean segmentation error of 0.28⁡mm (SD=0.24⁡mm) was reported for validation on a realistic bovine bone phantom immersed in solid gel.

The bone surfaces, obtained from the automatic segmentation of the ultrasound volumes, were used to register a high-resolution CT model of the tibia using iterative closest points. Twelve intraoperative registration experiments were performed using the same tibia phantom located at different positions on the experiment table. The target registration errors (TREs) of each experiment were calculated as the distances between all corresponding vertices in the registered CT mesh (*M*_CT‐intraop_) and a reference CT mesh (*M*_ct‐ref_). Table 3 shows a maximum mean TRE of 1.541⁡mm (SD=0.229⁡mm) for experiment two. The overall mean TRE for the twelve experiments was 0.64⁡mm which is smaller than other TREs previously reported: in the work of Beek et al. [22], was reported a mean TRE of 3.32⁡mm (SD=1.41⁡mm) for three alumina beads of 3.0⁡mm of diameter, which includes the uncertainty to locate the exact center of the alumina beads; Penney et al. [18] reported a RMS TRE of 2.4⁡mm or less for 3 registration experiments on the left and right femurs of cadavers.

Figure 6 shows the target registration errors (TREs) illustrated in a false color scale, for each point on the surface of the CT. As expected, smaller registration errors are obtained when large areas of the tibia phantom are scanned with the ultrasound probe. The lower row of Figure 6 shows three cases with mean target registration errors smaller than 0.24 mm.

The shape of the diaphysis (i.e., the middle section) in long bones is very similar along the bone. This makes it difficult to achieve an accurate registration if the scanned area contains only the diaphysis of the bone. In order to obtain better accuracy in the registration process, it is advisable to include part of the epiphysis (i.e., ends of the long bone) into the scan since the epiphysis contains features that can be captured with ultrasound images.  Figure 6 shows that when the scanned areas do not contain part of the epiphysis, the registration error is larger.

The mean registration time including ultrasound volume reconstruction, surface segmentation, and ICP registration was approximately eight minutes. All computations were made on a Mac Pro with a 2.8 GHz Quad Core Intel Xeon processor and 16 GB of RAM.

## 4. Conclusions

Fast and accurate intraoperative registration is a critical stage of most computer-assisted orthopedic surgery (CAOS) systems. The use of intraoperative ultrasound for image-guided registration in CAOS has several advantages: low cost, no exposure to ionizing radiation, and compact size. However, the accurate registration of CT and ultrasound of bones is still a research challenge due to the low signal to noise ratio of ultrasound imaging and its incapability to penetrate bones.

A new method for the intraoperative registration of preoperative CT volumes of long bones was reported in this work. The method is based on the automatic 3D segmentation of the bone cortical in 3D freehand ultrasound imaging which, in turn, enables the acquisition of large bone sections. The method is able to segment the bone surface in large ultrasound volumes with an overall mean segmentation error of 0.21⁡mm (SD=0.061⁡mm). The approximate segmentation time per volume was 1 min (for volumes of 850 × 850 × 850 voxels). The corresponding preoperative CT was manually segmented, and both meshes (ultrasound and CT) were accurately registered using iterative closest points. Accurate registration was achieved, with minimum user interaction. The maximum TRE was under 1.55⁡mm, and the mean maximum TRE was 0.64⁡mm.

It has been observed that long bones have very similar surface shapes in completely different locations. A conventional nonnavigated 3D ultrasound probe can only scan a small part of the bone. This makes 3D freehand ultrasound as an optimal choice for intraoperative registration of long bones since large parts of the bone can be scanned and automatically segmented with the method reported. After the acquisition of the 3D ultrasound images, the total processing time was approximately 8 min, with 5 min. for volume reconstruction, 1 min. for bone segmentation, and 2 min. for CT registration. Ultrasound volume reconstruction is suitable for parallel implementations which can reduce significantly the total reconstruction times. Atesok et al. [5] reported that, on average, 14 min. of extra surgical time are added for 2D fluoroscopic navigation in fracture reduction surgery. This extra time is acceptable for the surgeons given the localization advantages of navigated instruments during minimally invasive procedures.

The image segmentation and registration methods reported here are suitable for minimally invasive surgery of long bones such as fracture reduction of the femur and tibia. Other arthroscopic surgical procedures such as total knee replacement will be subject to the possibilities to scan the surgical site with ultrasound imaging.

## Figures and Tables

**Table 1:** Basic conditions for geometric structure discrimination.

Structure	Relation condition
Surface	*λ*_3_ ≪ *λ*_2_≃*λ*_1_≃⁡0
Tube	*λ*_3_≃⁡*λ*_2_ ≪ *λ*_1_≃⁡0
Sphere	*λ*_3_≃⁡*λ*_2_≃⁡*λ*_1_ ≪ 0

**Table 2:** Segmentation errors of the twelve experiments performed. For each experiment, the maximum, mean, and standard deviation errors, as well as the number of mesh vertices resulting from the segmented ultrasound volume are shown.

Experiment number	Maximum (mm)	Mean (mm)	SD (mm)	Number of vertices
1	1.586	0.164	0.146	4.0E5
2	1.153	0.14	0.096	1.2E5
3	2.314	0.158	0.148	4.2E4
4	2.566	0.334	0.257	3.5E5
5	2.064	0.253	0.205	2.8E5
6	2.141	0.207	0.164	2.7E5
7	2.003	0.276	0.227	3.6E5
8	2.316	0.211	0.216	2.2E5
9	4.292	0.267	0.224	3.7E5
10	1.216	0.159	0.119	1.1E5
11	1.232	0.199	0.169	1.1E5
12	1.489	0.15	0.135	2.0E5

**Table 3:** TRE of the twelve experiments performed.

Experiment number	Minimum (mm)	Maximum (mm)	Mean (mm)	SD (mm)
1	0.048	0.574	0.324	0.114
2	1.158	2.027	1.541	0.229
3	0.25	2.771	1.341	0.405
4	1.003	1.529	1.247	0.127
5	0.05	0.195	0.101	0.029
6	0.066	0.513	0.212	0.010
7	0.064	0.457	0.235	0.087
8	0.243	1.471	0.612	0.286
9	0.128	1.944	0.834	0.358
10	0.393	0.476	0.420	0.019
11	0.033	1.016	0.472	0.196
12	0.103	0.64	0.346	0.121
